# Translation and cultural adaptation of the DSM-5 Personality Inventory – Brief Form (PID-5-BF)

**DOI:** 10.1590/2237-6089-2019-0073

**Published:** 2020-05-27

**Authors:** Cleonice Zatti, Sérgio Eduardo Silva de Oliveira, Luciano Santos Pinto Guimarães, Vitor Crestani Calegaro, Silvia Pereira da Cruz Benetti, Fernanda Barcellos Serralta, Lucia Helena Machado Freitas

**Affiliations:** 1 Programa de Pós-Graduação em Psiquiatria e Ciências do Comportamento Universidade Federal do Rio Grande do Sul Porto AlegreRS Brazil Programa de Pós-Graduação em Psiquiatria e Ciências do Comportamento, Universidade Federal do Rio Grande do Sul (UFRGS), Porto Alegre, RS, Brazil.; 2 Departamento de Psicologia Clínica Programa de Pós-Graduação em Psicologia Clínica e Cultura Universidade de Brasília BrasíliaDF Brazil Departamento de Psicologia Clínica e Programa de Pós-Graduação em Psicologia Clínica e Cultura, Universidade de Brasília (UnB), Brasília, DF, Brazil.; 3 Hospital de Clínicas de Porto Alegre Porto AlegreRS Brazil Hospital de Clínicas de Porto Alegre (HCPA), Porto Alegre, RS, Brazil.; 4 Departamento de Neuropsiquiatria Universidade Federal de Santa Maria Santa MariaRS Brazil Departamento de Neuropsiquiatria, Universidade Federal de Santa Maria (UFSM), Santa Maria, RS, Brazil.; 5 Programa de Pós-Graduação em Psicologia Universidade do Vale do Rio dos Sinos São LeopoldoRS Brazil Programa de Pós-Graduação em Psicologia, Universidade do Vale do Rio dos Sinos (UNISINOS), São Leopoldo, RS, Brazil.

**Keywords:** Adaptation of instrument, evaluation in mental health, pathological personality traits

## Abstract

**Introduction:**

The Personality Inventory for the DSM-5 – Brief Form (PID-5-BF) – is an instrument for assessment of the five pathological personality traits from the Diagnostic and Statistical Manual of Mental Disorders, 5th edition (DSM-5) alternative model of personality disorders.

**Objectives:**

To determine the psychometric properties of the version of the PID-5-BF translated and adapted to Brazilian Portuguese.

**Methods:**

The process of translating and cross-culturally adapting the text was carried out by independent translators and the resulting version was administered to 176 patients in two hospitals in Rio Grande do Sul. The internal structure was tested by means of confirmatory factor analysis. Evidence of reliability was tested by examining the internal consistency of the scales and their convergent and concurrent validity with other methods of psychopathology.

**Results:**

The five factors were replicated in the present sample with adequate indicators of fit of the data to the model. Appropriate reliability coefficients for the scales and evidence of validity were observed, indicating the clinical usefulness of the PID-5-BF in the Brazilian context.

**Conclusion:**

The psychometric properties of PID-5-BF proved satisfactory in an initial sample of Brazilians.

## Introduction

The fifth edition of the Diagnostic and Statistical Manual of Mental Disorders (DSM-5)^1^ presents a new hybrid model for diagnosing personality disorders. The model includes a dimensional understanding of personality pathologies combined with a categorical perspective. In other words, patients can be classified into specific diagnostic categories based on a dimensional evaluation of the level of personality functioning (Criterion A) and of the pathological personality trait profiles (Criterion B).^1 , 2^ This dimensional model was developed in response to the limitations of the pure categorical model, which resulted, for example, in classification of a broad range of symptomatic heterogeneity into single diagnostic categories and a high prevalence of diagnosis of personality disorders not otherwise specified, as seen in numerous studies.^3 - 5^

This new diagnostic model, with its criterion B, brought in an empirical model based on pathological personality traits. The results of initial studies indicated five broad factors that describe an individual’s main maladaptive behaviors, beliefs, thoughts and feelings.^3^ These factors are negative affectivity, detachment, antagonism, disinhibition, and psychoticism, and they can be assessed using the Personality Inventory for the DSM-5 (PID-5).^3^ This instrument is currently available in five formats: 1) the complete self-report version (PID-5), composed of 220 items^3^ ; 2) the other informant version (PID-5-IRF), containing 218 items^6^ ; 3) the self-report short form (PID-5-SF), containing 100 items^7^ ; 4) the self-report brief form (PID-5-BF), containing 25 items^8^ ; and 5) the self-report brief form plus (PID-5-BF+), containing 36 items to assess 18 facets organized into six broad factors covering the DSM-5 and International Classification of Diseases, 11th revision (ICD-11) personality pathology models.^9 , 10^ The sixth factor is related to the anankastic/compulsivity trait. The different versions of the PID-5 serve specific needs of clinicians and researchers. The focus of the current study is the Personality Inventory for DSM-5 – Brief Form (PID-5-BF), which is well-known for being a quick tool for evaluating the five pathological personality traits according to the DSM-5 alternative model and is seen as a useful tool to aid decision-making in clinical settings.^4 , 5^

As a measure for tracking personality pathology, the PID-5-BF has a number of potentially attractive characteristics, such as rapid verification of pathological personality traits, demanding little time to answer the questionnaire, and little time for the clinician to present results. Studies have shown that the PID-5-BF has adequate psychometric properties.^2 , 5 , 11 - 13^ In fact, the literature indicates a strong correspondence between scores on the complete version of the PID-5 and scores on the brief form (PID-5-BF).^5 , 11 , 12^ All of its factors have presented satisfactory indicators of reliability according to Cronbach’s alpha and indicators of validity, showing a consistent nomological network between the PID-5-BF factor scores and scores from measures of psychiatric symptoms,^2 , 5 , 11^ the five-factor personality model,^2 , 5 , 14^ and other personality pathology models.^11 - 13^

Considering the clinical and scientific utility of the PID-5-BF for estimating pathological personality traits, the present study aims to determine the initial psychometric properties of a Brazilian translated and adapted version of this instrument. The specific objectives were 1) to analyze the internal structure of the PID-5-BF, to verify whether the five factors would be detected in a Brazilian sample, 2) to investigate the degree of reliability of the scales, and 3) to examine the validity of the PID-5-BF scores by analyzing their associations with a selection of psychiatric syndromes. To this end, two studies were conducted. The objective of the first was to conduct the translation to Brazilian Portuguese and cross-cultural adaptation of the items in the PID-5-BF, according to international recommendations on the cultural adaptation of instruments.^6 , 7^ The second study investigated the psychometric properties of the PID-5-BF in a sample of patients from two hospitals in the southern region of Brazil.

## Study I: Translation and cross-cultural adaptation of the PID-5-BF for Brazil

The literature consistently recommends that any psychological instrument should undergo a rigorous process of translation and cultural adaptation before it is used in a country different from its origin.^15^ The procedures of translation and adaptation guarantee that the instrument has semantic, idiomatic, experimental, cultural, and conceptual equivalence with the original version. The objective of the present study was to translate and adapt the items of the PID-5-BF into Brazilian Portuguese, in order to guarantee its technical and clinical quality in Brazil. The authors of this article were granted authorization by Editora Artmed, which holds the exclusive rights for translation of the DSM-5 and supplementary material in Brazil.

### Method

**Figure 1:**
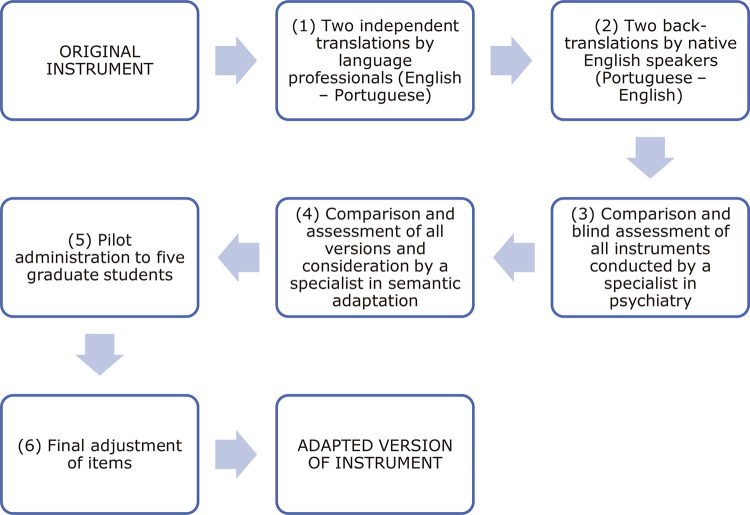
Flowchart of procedures for translation and cross-cultural adaptation of the Personality Inventory for the DSM-5 – Brief Form (PID-5-BF)

Translation and cross-cultural adaptation were conducted in six steps, which are recommended in the main guidelines for cross-cultural adaptation of instruments. Figure 1 contains a flowchart illustrating the steps taken to translate and cross-culturally adapt the PID-5-BF for the Brazilian language and culture.

Initially, (1) two translations were independently produced by two Brazilian professionals with broad knowledge of the English language. Next, (2) two professors of English, both of whom are natives of the United States but are living in Brazil and are fluent in Portuguese, carried out a back-translation from Brazilian Portuguese to English. The English versions were then sent to a fifth professional (3), an experienced psychiatrist, fluent in English and familiar with the PID-5-BF construct, who compared and evaluated the original items and the items from the two back-translated versions. This was a blind evaluation, since the professional did not know which items were from the original and which had been back-translated. Next, (4) the authors of the present study evaluated the versions in Portuguese, the original version, and the back-translated versions. All of them were compared and systematically reviewed, discussing the corresponding meanings in the Brazilian culture. The authors then constructed a Brazilian Portuguese version of the PID-5-BF, taking into account the information collected in the previous steps. This last version was then administered to five graduate students in psychiatry (5). The objective of this stage was to verify whether patients would be able to understand the instructions and the items of the Brazilian Portuguese version of the PID-5-BF, and whether they would be able to properly respond to the items (response process). Finally, the necessary adjustments and revisions were made, to conclude the final version of the instrument in Brazilian Portuguese (6).

### Results and discussion

Very few cultural adaptations were necessary. Three items were adapted in order to replace unfamiliar words. For example, the English expression “Zone Out” presented in the 23rd item was translated as “I go off air,” which is a Brazilian expression that imparts the same meaning as the English expression and is familiar to Brazilians. In addition to employing procedures to maintain semantic equivalence between the original and translated versions of the instruments, operational equivalence was also prioritized. Therefore, the operational characteristics of the original instrument, such as the same number of questions and the same four response options, were maintained.

Based on the procedures used and the results found, we believe that the two versions of the PID-5-BF (i.e., the original and the Brazilian) are semantically and operationally equivalent. It is understood, therefore, that the instrument can be used in Brazil and that possible differences between the results observed in the two cultures (the United States and Brazil) will not be due to semantic differences between the instrument versions, but rather due to cultural differences in expression of pathological personality traits.

Finally, it is worth mentioning that when this study began, the main authors of the current study were not aware that other versions of the PID-5 were under development.^16 , 17^ The authors compared the versions of the instrument and found that they were very similar. Once the authors became acquainted with the Brazilian Portuguese version of the full PID-5,^17^ they decided to use the items from the full version, to maintain continuity between the two versions of the instruments (i.e., the full and the brief forms). The Brazilian version used in this study can be requested from the author by email.

## Study II: Psychometric study of the Brazilian version of the PID-5-BF

Notwithstanding the need to conduct a process of cross-cultural adaptation of an instrument to guarantee its comparability in other cultures, it is also necessary to evaluate its psychometric properties in these new populations. The degree of precision of its items for estimating latent traits in the new target population must be verified and the instrument’s validity must also be examined. The aim of the current study was therefore to verify the validity and reliability of the Brazilian version of the PID-5-BF.

### Method

#### Participants

A total of 176 individuals were recruited at two public hospitals in the State of Rio Grande do Sul and participated in this study. The hospitals involved were the Hospital de Pronto Socorro de Porto Alegre, southern Brazil (HPS), and the Hospital Universitário de Santa Maria (HUSM), Santa Maria, southern Brazil. At the HPS, data were collected on 84 people, either inpatients or people being treated in the emergency room, and at the HUSM the participants were 92 subjects being treated at the Integrated Care Center for Accident Victims (Centro Integrado de Atendimento às Vítimas de Acidentes [CIAVA]).

#### Instruments

**Sociodemographic questionnaire.** A questionnaire was drawn up to obtain general information about the participants, such as sex, age, years of study, income, marital status, and occupation.

**Personality Inventory for the DSM-5 - Brief Form (PID-5-BF).** The instrument comprises 25 items, with a four-point Likert response scale (0 = never to 3 = always), evaluating the five pathological personality traits, namely, negative affectivity, detachment, antagonism, disinhibition and psychoticism. As previously reported, this instrument presents adequate psychometric properties.^2 , 5 , 11 - 14^

**Mini-International Neuropsychiatric Interview – Plus (MINI).** This is a brief and standardized diagnostic interview lasting 15 to 30 minutes that evaluates the criteria for the main mental disorders described in DSM-IV. The Brazilian version was used, since it has shown adequate psychometric proprieties. The coefficients for interexaminer agreement and test-retest reliability were above 0.75 for all diagnoses except current manic episode (0.35).^18^

#### Procedures

Data collection at the HPS was conducted by a psychologist-researcher with experience with hospitalized patients. While evaluating the hospitalized patients’ conditions, the researcher also explained to them the objectives of the study and presented them with a copy of the free and informed consent form. Once the inclusion criteria had been verified and the participant had given consent for enrollment, the sociodemographic questionnaire with questions covering general information was then administered, followed by the PID-5-BF, to evaluate pathological personality traits and, finally, the MINI, to assess mental disorders.

Data were collected at the HUSM by a group of researchers at the hospital and participants were referred from the CIAVA. The researchers explained the objectives of the research and presented the Free and Informed Consent Form. Once the inclusion criteria had been verified and the participant had given consent for enrollment, and after each patient had been seen by the outpatient service, they were then taken to a private consultation room and the instruments were administered, following the same order of administration as at the HPS.

#### Data analysis

Initially, we explored the sociodemographic variables to describe participants’ characteristics. Next, confirmatory factor analysis (CFA) was conducted to examine the internal structure of the Brazilian version of the PID-5-BF. MPlus software (version 7.40) was used for this analysis. The fit to the data was analyzed using the comparative fit index (CFI), Tucker-Lewis index (TLI), root mean square error of approximation (RMSEA), and weighted root mean square residual (WRMR). The cut-off criteria for acceptable model fit are ≥ 0.90 for CFI/TLI, < 0.08 for RMSEA, and < 1.0 for WRMR.^19 - 23^

Reliability was examined in terms of internal consistency coefficients. Cronbach’s alpha and McDonald’s omega were calculated.^24 , 25^ Coefficients for average variance extracted (AVE) and composite reliability (CR) were also used.^19 , 23 , 26^ Calculations to determine CR and AVE are based on the estimated parameters of the CFA. Values for AVE equal to or greater than 0.50 are considered indicative of adequate model fit.^19 , 23^ The recommendation for CR is values < 0.70 or even < 0.60.^26^

Validity was investigated by measuring correlations between the PID-5-BF factors, the MINI suicide risk scale, and the total number of diagnoses identified by the MINI. These two external criteria were chosen because they were variables registered in a non-binary way in the database. Our hypothesis, based on the results of previous research, was that pathological personality traits are related to suicidal behavior^27^ and to the number of mental disorder diagnoses.^28^ We also examined the PID-5-BF score’s ability to differentiate patients with different diagnostic categories, using the Mann-Whitney U-Test. Finally, Hierarchical Logistic Regression models were constructed to verify the individual contribution made by PID-5-BF factors to prediction of different mental disorder categories. Models were constructed in two steps, by forced entry. In the first step, the variables sex, age, and years of study were added and then the PID-5-BF factor scores were added in the second step. The models’ fit indexes were determined and residuals were analyzed.

#### Ethical aspects

All participants were given the necessary explanations regarding the procedures involved in the research. The study is registered under ethics commission submission protocol number 44823315.1.0000.5327. It was approved by the research ethics commissions of HCPA, of the Porto Alegre Municipal Health Department (certificate 1.180.317), and of Universidade Federal de Santa Maria (certificate 39906414.8.0000.5346).

## Results

Table 1 presents the sociodemographic data for the sample, comprising individuals recruited at the HPS and the HUSM. There was a greater proportion of participants in psychiatric services at the HUSM than at the HPS. Additionally, the participants from the HUSM tended to be younger, but with more years in education, had lower family income, and more occupational activity (i.e. working or studying), and had fewer stable intimate relationships, than the participants from HPS.

**Table 1:** Sociodemographic data for the sample

	Total	HPS	HUSM	p
Sex*				
Men	87 (49.4)	39 (46.4)	48 (52.2)	0.542
Women	89 (50.6)	45 (53.6)	44 (47.8)	
Age (years)^†^				
Mean (SD)	32.7 (11.5)	35.6 (12.8)	30.1 (9.5)	0.019
Median [q1;q3]	29 [23;41]	34 [23;44.75]	26 [23;35]	
Years of study,^†^ median [q1;q3]	13 [9;15]	10 [7;12.3]	14 [13;16]	<0.001
Family income (R$),^†^ median [q1;q3]	2,000 [1,000;3,500]	2,000 [1,260;3,650]	1,600 [800;3,200]	0.027
Marital status*				
W/o steady partner	85 (48.6)	30 (35.7)	**55 (60.4)**	
With steady partner	70 (40)	**41 (48.8)**	29 (31.9)	
Widowed	3 (1.7)	1 (1.2)	2 (2.2)	
Separated	17 (9.7)	**12 (14.3)**	5 (5.5)	
Occupation*				
Unemployed	33 (18.9)	**22 (26.5)**	11 (12.0)	< 0.001
Employed	94 (53.7)	37 (44.6)	**57 (62.0)**	
Retired	6 (3.4)	**6 (7.2)**	0 (0)	
On leave	16 (9.1)	9 (10.8)	7 (7.6)	
Self-employed	6 (3.4)	**6 (7.2)**	0 (0)	
Student	20 (11.4)	3 (3.6)	**17 (18.5)**	
Psychiatric history*				
No	132 (75)	62 (73.8)	70 (76.1)	0.862
Yes	44 (25)	22 (26.2)	22 (23.9)	
Psychiatric treatment*				
No	117 (66.5)	**73 (86.9)**	44 (47.8)	< 0.001
Yes	59 (33.5)	11 (13.1)	**48 (52.2)**	
Psychological treatment*				
No	125 (71.8)	**68 (81.0)**	57 (63.3)	0.016
Yes	48 (27.6)	16 (19.0)	**32 (35.6)**	
Treatment*				
No	103 (58.9)	**62 (73.8)**	41 (45.1)	< 0.001
Yes (psychiatric or psychological)	35 (20)	5 (6.0)	**30 (33.0)**	
Yes (psychiatric and psychological)	37 (21.1)	17 (20.2)	20 (22.0)	

Data presented as n (%), unless otherwise specified.

HPS = Hospital de Pronto-Socorro de Porto Alegre; HUSM = Hospital Universitário de Santa Maria; q1;q3 = first and third quartiles; SD = standard deviation.

Bold type indicates that adjusted standardized residuals are greater than or equal to 1.96.

* Categorical variables analyzed using the chi-square test.

^†^ Quantitative variables analyzed using the Mann-Whitney test.

The internal structure of PID-5-BF was tested using CFA and the results are shown in Table 2 . Acceptable fit indexes were achieved for CFI (0.91), TLI (0.90), and RMSEA (0.07; 90% confidence interval = 0.06-0.08). The chi-square statistic value was 493.777 ( *gl* = 265; p < 0.001) and the WRMR was 1.084. Factor loadings were all above 0.50 except for item 13 in the detachment factor (λ = 0.43).

**Table 2:** Confirmatory factor analysis of the PID-5-BF, reliability coefficients and correlations among factors

Item	Negative affectivity	Detachment	Antagonism	Disinhibition	Psychoticism	Residual variance
8	0.525	-	-	-	-	0.557
9	0.642	-	-	-	-	0.317
10	0.721	-	-	-	-	0.446
11	0.833	-	-	-	-	0.202
15	0.710	-	-	-	-	0.363
4	-	0.893	-	-	-	0.484
13	-	0.427	-	-	-	0.411
14	-	0.703	-	-	-	0.725
16	-	0.727	-	-	-	0.588
18	-	0.708	-	-	-	0.481
17	-	-	0.796	-	-	0.306
19	-	-	0.632	-	-	0.370
20	-	-	0.583	-	-	0.818
22	-	-	0.672	-	-	0.505
25	-	-	0.716	-	-	0.496
1	-	-	-	0.666	-	0.471
2	-	-	-	0.826	-	0.367
3	-	-	-	0.744	-	0.498
5	-	-	-	0.798	-	0.601
6	-	-	-	0.718	-	0.660
7	-	-	-	-	0.767	0.348
12	-	-	-	-	0.794	0.548
21	-	-	-	-	0.808	0.472
23	-	-	-	-	0.727	0.276
24	-	-	-	-	0.851	0.487
AVE	0.481	0.501	0.467	0.566	0.625	
CR	0.862	0.816	0.822	0.844	0.880	
Omega [95%CI]	0.765 [0.703-0.826]	0.752 [0.684-0.820]	0.519 [0.258-0.780]	0.811 [0.760-0.863]	0.820 [0.743-0.896]	
Alpha [95%CI]	0.758 [0.694-0.821]	0.754 [0.685-0.823]	0.560 [0.409-0.784]	0.805 [0.750-0.860]	0.823 [0.747-0.900]	
Negative affectivity	-	-	-	-	-	
Detachment	0.730	-	-	-	-	
Antagonism	0.547	0.767	-	-	-	
Disinhibition	0.722	0.739	0.660	-	-	
Psychoticism	0.777	0.823	0.684	0.764	-	

95%CI = 95% confidence interval; AVE = average variance extracted; CR = composite reliability; PID-5-BF = Personality Inventory for the DSM-5 – Brief Form.

The reliability of the scales was estimated using several strategies (see results in Table 2 ). The results were adequate for all scales except antagonism. All scales had adequate results for the CR and AVE coefficients.

The validity of the PID-5-BF was analyzed by correlating its scores with the participants’ degree of suicide risk and with the total number of diagnoses of mental disorders according to the MINI-Plus.^18^Table 3 presents the correlation coefficients obtained by these criteria. The detachment and disinhibition factors presented the strongest correlation coefficients with suicide risk. The strongest correlations with the total number of diagnoses were for detachment, negative affectivity, and disinhibition.

**Table 3:** Correlation of the PID-5-BF scales with degree of suicide risk and number of diagnoses according to the MINI-Plus

Domains of the PID-5-BF	Suicide risk, rho (p)	Number of diagnoses, rho (p)
Negative affectivity	0.367 (< 0.001)	0.519 (< 0.001)
Detachment	0.498 (< 0.001)	0.528 (< 0.001)
Antagonism	0.283 (< 0.001)	0.303 (< 0.001)
Disinhibition	0.487 (< 0.001)	0.501 (< 0.001)
Psychoticism	0.384 (< 0.001)	0.476 (< 0.001)
PID-5-BF total	0.514 (< 0.001)	0.635 (< 0.001)

MINI-Plus = Mini-International Neuropsychiatric Interview – Plus; PID-5-BF = Personality Inventory for the DSM-5 – Brief Form.

Another investigation of validity compared the scores of the PID-5-BF between participants who either did or did not fulfill the criteria for a mental disorder according to the MINI-Plus. Table 4 presents the results obtained. For this analysis, disorder categories were grouped as follows: mood disorders included depressive disorder, dysthymia, and bipolar disorder; anxiety disorders included social phobia, agoraphobia, panic disorders, and generalized anxiety disorder; and eating disorders included bulimia and anorexia nervosa. Obsessive-compulsive disorder, post-traumatic stress disorder, and psychotic syndrome were not grouped into any specific categories.

**Table 4:** Comparison of PID-5-BF scores by diagnoses identified by the MINI-Plus

	Mood disorders			Anxiety disorders			Obsessive-compulsive disorder		

	No (n = 113) q2 [q1;q3]	Yes (n = 55) q2 [q1;q3]	p	No (n = 107) q2 [q1;q3]	Yes (n = 61) q2 [q1;q3]	p	No (n = 159) q2 [q1;q3]	Yes (n = 9) q2 [q1;q3]	p
Negative affectivity	1.2 [0.8;1.6]	1.6 [1.2;2.4]	< 0.001	1.2 [0.8;1.6]	1.8 [1.2;2.4]	< 0.001	1.2 [0.8;1.8]	2.4 [2.0;3.0]	< 0.001
	Z = -4.994; ES = -0.39			Z = -5.315; ES = -0.41			Z = -3.865; ES = -0.30		
Detachment	0.4 [0.2;0.8]	1.2 [0.8;1.8]	< 0.001	0.6 [0.2;1.0]	1.0 [0.4;1.6]	0.005	0.6 [0.2;1.0]	1.8 [1.4;2.4]	0.011
	Z = -6.779; ES = -0.52			Z = -3.815; ES = -0.30			Z = -4.198; ES = -0.32		
Antagonism	0.4 [0.2;0.6]	0.8 [0.4;1.2]	< 0.001	0.4 [0.2;0.6]	0.6 [0.2;1.2]	< 0.001	0.4 [0.2;0.8]	1.2 [0.8;1.6]	< 0.001
	Z = -4.162; ES = -0.32			Z = -2.825; ES = -0.22			Z = -2.537; ES = -0.20		
Disinhibition	0.6 [0.3;0.8]	1.2 [0.6;2.0]	< 0.001	0.6 [0.3;1.0]	1.0 [0.6;1.8]	< 0.001	0.6 [0.4;1.0]	2.2 [2.0;2.2]	< 0.001
	Z = -5.001; ES = -0.39			Z = -4.430; ES = -0.34			Z = -4.541; ES = -0.35		
Psychoticism	0.2 [0.0;0.6]	0.8 [0.2;1.4]	< 0.001	0.2 [0.0;0.6]	0.6 [0.2;1.4]	< 0.001	0.4 [0.0;0.8]	1.4 [1.0;3.0]	< 0.001
	Z = -4.473; ES = -0.35			Z = -4.964; ES = -0.38			Z = -3.732; ES = -0.29		
PID-5-BF total	0.6 [0.4;0.8]	1.2 [0.8;1.7]	< 0.001	0.6 [0.4;0.8]	1.1 [0.7;1.5]	< 0.001	0. 7 [0.5;1.0]	1.9 [1.7;2.3]	< 0.001
	Z = -6.992; ES = -0.54			Z = -5.793; ES = -0.45			Z = -4.542; ES = -0.35		

	**Post-traumatic stress disorder**			**Psychotic syndrome**			**Eating disorders**		
	
	**n = 136**	**n = 32**	**p**	**n = 152**	**n = 16**	**p**	**n = 165**	**n = 3**	**p**

Negative affectivity	0.6 [0.2;1.0]	1.2 [0.6;1.6]	< 0.001	0.6 [0.2;1.0]	1.6 [1.3;2.3]	< 0.001	0.6 [0.2;1.2]	2.4 [0.8;2.4]	0.035
	Z = -3.238; ES = -0.25			Z = -4.834; ES = -0.37			Z = -2.285; ES = -0.18		
Detachment	0.4 [0.2;0.8]	0.6 [0.3;1.2]	0.027	0.4 [0.2;0.8]	1.1 [0.6;1.5]	< 0.001	0.4 [0.2;0.8]	1.4 [0.4;3.0]	0.079
	Z = -3.593; ES = -0.28			Z = -4.302; ES = -0.33			Z = -2.106; ES = -0.16		
Antagonism	0.6 [0.4;1.2]	1.0 [0.6;1.7]	0.006	0.6 [0.4;1.0]	2.2 [1.9;2.5]	< 0.001	0.6 [0.4;1.2]	2.2 [1.6;2.6]	0.011
	Z = -2.205; ES = -0.17			Z = -3.768; ES = -0.29			Z = -1.757; ES = -0.14		
Disinhibition	0.4 [0.0;0.6]	0.7 [0.2;1.4]	< 0.001	0.4 [0.0;0.6]	1.4 [0.8;2.8]	< 0.001	0.4 [0.0;0.8]	1.2 [0.6;3.0]	0.045
	Z = -2.775; ES = -0.21			Z = -5.059; ES = -0.39			Z = -2.554; ES = -0.20		
Psychoticism	0.6 [0.4;0.9]	1.1 [0.8;1.4]	< 0.001	0.6 [0.4;1.0]	1.8 [1.3;2.1]	< 0.001	0.7 [0.5;1.1]	1.9 [1.1;2.7]	0.016
	Z = -3.676; ES = -0.28			Z = -4.583; ES = -0.35			Z = -2.004; ES = -0.16		
PID-5-BF total	1.2 [0.8;1.6]	1.8 [1.3;2.2]	0.001	1.2 [0.8;1.6]	2.4 [1.7;2.8]	< 0.001	1.4 [0.8;1.8]	2.0 [2.0;3.0]	0.022
	Z = -4.166; ES = -0.32			Z = -5.443; ES = -0.42			Z = -2.399; ES = -0.19		

ES = effect size; MINI-Plus = Mini-International Neuropsychiatric Interview – Plus; PID-5-BF = Personality Inventory for the DSM-5 – Brief Form; q1 = first quartile (25%); q2 = second quartile (50%); q3 = third quartile (75%); Z = Z score.

The main differences between participants with or without mood disorders were identified in scores for the detachment and negative affectivity factors, respectively. The anxiety disorders group differed mainly in terms of the negative affectivity, psychoticism, and disinhibition factors. For the obsessive-compulsive disorder category, disinhibition, detachment, and negative affectivity factors exhibited the main differences. The post-traumatic stress disorder category was differentiated mainly by the negative affectivity factor. The factors that differentiated groups of participants with or without psychotic syndrome, were disinhibition, negative affectivity, and psychoticism. The greatest differences for the eating disorders category were identified in the disinhibition, negative affectivity, and detachment factors.

Finally, the personality characteristic most associated with mood disorders was detachment. for anxiety disorders, psychoticism was the variable that showed an effect between groups. Psychoticism increased the chances of having anxiety disorder by almost 3.2. The mental disorder psychotic syndrome was associated with the disinhibition variable. This finding should be interpreted with caution, because the confidence interval is wider than the others. Detachment was the most prominent factor in the analyses of screening positive for any disorder. Having Detachment increased the chance of being diagnosed with a mental disorder by 3.2. After controlling for sex and years of study, none of the personality variables were associated with PTSD (see Table 5 ). The obsessive-compulsive disorder and eating disorders categories were not analyzed because fewer than 10 cases screened positive for them, which meant that analysis was unfeasible.

**Table 5:** Hierarchical logistic regression model using the PID-5-BF total score as predictor for diagnosis of some of the mental disorders identified by the MINI-Plus

	OR	95%CI	p
Mood disorders (yes = 54 vs. no = 112)			
Male sex	0.387	0.153-0.977	0.044
Age	1.030	0.992-1.070	0.118
Years of study	0.935	0.842-1.038	0.206
Negative affectivity	1.035	0.484-2.214	0.930
Detachment	5.142	2.083-12.690	0.000
Antagonism	1.975	0.700-5.575	0.199
Disinhibition	2.036	0.884-4.689	0.095
Psychoticism	1.074	0.437-2.635	0.877
Post-traumatic stress disorder (yes = 31 vs. no = 135)			
Male sex	0.128	0.040-0.412	0.001
Age	1.003	0.963-1.045	0.871
Years of study	1.148	1.018-1.295	0.024
Negative affectivity	1.285	0.549-3.009	0.563
Detachment	1.638	0.692-3.880	0.262
Antagonism	2.492	0.969-6.404	0.058
Disinhibition	1.171	0.501-2.739	0.715
Psychoticism	1.405	0.593-3.328	0.440
Anxiety disorders (yes = 61 vs. no = 105)			
Male sex	0.383	0.171-0.857	0.020
Age	0.993	0.960-1.027	0.670
Years of study	1.022	0.929-1.125	0.653
Negative affectivity	1.990	0.969-4.085	0.061
Detachment	0.874	0.404-1.892	0.733
Antagonism	1.178	0.474-2.924	0.725
Disinhibition	1.786	0.840-3.799	0.132
Psychoticism	3.191	1.281-7.948	0.013
Psychotic syndrome (yes = 16 vs. no = 150)			
Male sex	0.691	0.118-4.064	0.683
Age	0.923	0.844-1.008	0.075
Years of study	0.838	0.658-1.067	0.152
Negative affectivity	2.691	0.619-11.690	0.187
Detachment	1.041	0.230-4.717	0.958
Antagonism	1.335	0.262-6.810	0.729
Disinhibition	5.521	1.388-21.966	0.015
Psychoticism	2.607	0.726-9.356	0.142
Any disorder (yes = 88 vs. no = 78)			
2Male sex	0.212	0.095-0.475	0.000
Age	1.012	0.978-1.048	0.495
Years of study	0.974	0.887-1.071	0.591
Negative affectivity	1.701	0.820-3.528	0.153
Detachment	3.239	1.366-7.681	0.008
Antagonism	0.212	0.095-0.475	0.000
Disinhibition	1.012	0.978-1.048	0.495
Psychoticism	0.974	0.887-1.071	0.591

95%CI = 95% confidence interval; OR = odds ratio; p = statistical probability value; PID-5-BF = Personality Inventory for the DSM-5 – Brief Form.

## Discussion

The present study aimed to present the procedures used for the cross-cultural adaptation of the PID-5-BF for the Brazilian culture and to investigate its psychometric properties in a sample of patients from two public hospitals in the southern region of Brazil. In general, the results indicated that the Brazilian version of the PID-5-BF has semantic and operational equivalence with the original version, guaranteeing the possibility of comparing intercultural data. Additionally, adequate psychometric properties were observed, suggesting that the Brazilian version of the PID-5-BF is a valid and reliable method for estimating pathological personality traits according to the DSM-5 model. Next, certain results found in the present study are discussed in detail which may contribute to understanding the DSM-5 model of pathological personality traits.

### Replicability of the five-factor structure of the DSM-5 pathological personality traits model

The five factors of pathological personality structure were replicated in the present study. The restrictive methodological approach, that is, the CFA used here, shows that the data had an acceptable fit to the theoretical model. Previous studies using PID-5-BF have empirically recovered the five factors.^2 , 5 , 11 - 14^ This consistency across different studies and samples, including different cultures, such as the French,^2^ the Italian,^13^ the European Portuguese,^5^ the Argentinian,^14^ and the Danish^12^ cultures, may suggest the universality of the pathological traits of the personality.

The residuals observed in our study (WRMR) remained a little above what we expected (> 1.00). We believe that this may be due to the restrictiveness of the model tested, because it makes cross-loadings impossible even though the DSM-5 pathological personality model is complex and dynamic.^3^ In any case, the results found here indicate that the Brazilian data adequately fit the model.

### Reliability of the PID-5-BF for estimating pathological personality traits

In the present study, several different methods for estimation of internal consistency were used to enable a careful examination of the adequacy of the items for representing latent traits. Detachment, disinhibition, and psychoticism factors had adequate coefficients for all four methods used. The negative affectivity factor had adequate coefficients for three of the four methods, but the antagonism factor had just one adequate coefficient. Other studies have reported slightly more robust reliability coefficients for antagonism (0.62^5^; 0.68^2^), but none have reported coefficients higher than 0.60. We therefore suggest investigating the reliability of this factor in other samples, preferably larger than the one studied here. Nevertheless, the CR coefficient suggests the factor has adequate reliability.^26^

### Validity of the Brazilian version of the PID-5-BF

The validity of the PID-5-BF was examined in different ways. Initially, the correlations of the PID-5-BF scores with the level of suicide risk and with the number of mental disorders screened positive by the MINI-Plus were evaluated. The literature points to a relationship between suicidal behavior and the presence of personality pathologies.^29 , 30^ This relationship was especially evident for the detachment and disinhibition factors. In other words, traits of social isolation and constricted affect, together with compulsive and erratic behavior, tend to be associated with suicidal thoughts and behaviors. Social isolation is considered a factor that increases the risk of suicide,^31^ as is a failure in the capacity for premeditation, that is, of a person behaving without considering potential consequences of their action.^32^ People with impulsive traits, a prior attempt, behaving in a non-planned manner, and together with the appearance of possible stressful life events, should be seen as at risk of the possibility of a possibly lethal suicide attempt.^33^ Furthermore, the level of disinhibition may be indicative of the speed with which a person moves from thought to action. Based on these findings, we suggest that clinicians evaluate the risk of suicide in patients who score high on the detachment and disinhibition factors, because, as described in the literature, these factors are related to suicidal behaviors and thoughts.^33 , 34^

Our results are intriguing, since the negative affectivity variable is usually a predictor of mental disorder, whereas in our analysis this factor had a negative result. The psychotic syndrome variable was associated with the disinhibition factor, and a group that was positive would have problems controlling behavior. The psychopathological status of anxiety disorders was associated with the psychoticism variable, and this group of people may present eccentric changes, perceptions, thinking, and behavior. The leading variable related to psychotic disorder was disinhibition and common characteristics within this framework are impulsivity and erratic behavior. Among the analyses for diagnosis of any disorder, the particularity most common was detachment which indicates that people with mental disorders have problems in their social relationships. It should be noted that our initial hypothesis was that the PID-5 variable psychoticism would be within the psychotic syndrome framework of the MINI-Plus. Psychoticism was present in the framework of anxiety disorders. What should be observed is that people with anxiety disorders have difficulty with reality, behavior and perception.

Another expectation of the present study was that PID-5-BF factors would correlate with the total number of mental disorders screened positive. New taxonomic models of mental disorders have incorporated pathological personality traits together with symptoms of mental disorders in order to examine the latent structure of pathological mental functioning.^35 - 37^ At the highest level of the hierarchy of this model, one finds a general factor of psychopathology characterized by an individual’s propensity to exhibit diffuse and varied disorders. It is therefore expected that the greater the number of mental disorders a person exhibits, the broader the diffusion of symptomatology and the greater the odds of exhibiting pathological personality traits.

Another strategy to verify the validity of the PID-5-BF was to compare scores obtained in different diagnostic categories. The disinhibition and negative affectivity factors discriminated between groups the most. These factors refer, in general, to a failure in behavioral and emotional control,^1^ and are common problems in the various different types of mental disorders.

### Limitations of the study

The present study was conducted with a sample of hospital patients who did not necessarily have any mental disorders. The sample comprised a small number of hospital patients from just two hospitals in the southern region of Brazil. Moreover, the majority of the disorders identified in the study were based on internalizing spectra, with only a few subjects showing disturbed thoughts and externalizing problems. It is also worth mentioning that the MINI-Plus was applied and scored by just one trained clinician and there was no opportunity to check the reliability of classifications. In the analyses of the MINI-Plus against the PID-5, the n(Positive) was low compared with n(Negative). Further investigation is required to obtain more robust results.

It is therefore not recommended that the results found here be generalized. Before this, new studies should be made using different samples and larger numbers of participants.

## Conclusion

The objective of the present study was to investigate the initial psychometric properties of the PID-5-BF in a Brazilian sample. The results indicate the instrument’s adequacy as a measure for identifying pathological personality traits among Brazilian patients in a hospital setting. The five-factor structure was confirmed in the present sample and there were satisfactory indicators of the scale’s reliability. We recommend further investigation of the reliability of the antagonism factor to clarify the scale’s adequacy for estimating this latent trait. The PID-5-BF scores were related to symptoms of different mental disorders, suggesting both the validity of the instrument and the theoretical proposition of a dimensional model of psychopathology.
